# A framework for developing a real-time lake phytoplankton forecasting system to support water quality management in the face of global change

**DOI:** 10.1007/s13280-024-02076-7

**Published:** 2024-09-20

**Authors:** Cayelan C. Carey, Ryan S. D. Calder, Renato J. Figueiredo, Robert B. Gramacy, Mary E. Lofton, Madeline E. Schreiber, R. Quinn Thomas

**Affiliations:** 1 Department of Biological Sciences, Virginia Tech, 926 West Campus Drive, Blacksburg, VA 24061 USA; 2 Center for Ecosystem Forecasting, Virginia Tech, 1015 Life Science Circle, Blacksburg, VA 24061 USA; 3 Department of Population Health Sciences, Virginia Tech, 205 Duck Pond Drive, Blacksburg, VA 24061 USA; 4 Department of Civil and Environmental Engineering, Duke University, Box 90287, Durham, NC 27708 USA; 5 Department of Electrical and Computer Engineering, University of Florida, 968 Center Drive, Gainesville, FL 32611 USA; 6 Department of Statistics, Virginia Tech, 250 Drillfield Drive, Blacksburg, VA 24061 USA; 7 Department of Geosciences, Virginia Tech, 926 West Campus Drive, Blacksburg, VA 24061 USA; 8 Department of Forest Resources and Environmental Conservation, Virginia Tech, 310 West Campus Drive, Blacksburg, VA 24061 USA

**Keywords:** Cyanobacteria, Cyberinfrastructure, Decision support, Forecast, Phytoplankton bloom, Water management

## Abstract

Phytoplankton blooms create harmful toxins, scums, and taste and odor compounds and thus pose a major risk to drinking water safety. Climate and land use change are increasing the frequency and severity of blooms, motivating the development of new approaches for preemptive, rather than reactive, water management. While several real-time phytoplankton forecasts have been developed to date, none are both automated and quantify uncertainty in their predictions, which is critical for manager use. In response to this need, we outline a framework for developing the first automated, real-time lake phytoplankton forecasting system that quantifies uncertainty, thereby enabling managers to adapt operations and mitigate blooms. Implementation of this system calls for new, integrated ecosystem and statistical models; automated cyberinfrastructure; effective decision support tools; and training for forecasters and decision makers. We provide a research agenda for the creation of this system, as well as recommendations for developing real-time phytoplankton forecasts to support management.

## Introduction

Water quality in lakes and reservoirs around the globe is increasingly at risk due to climate and land use change (e.g., Jane et al. 2021; Caretta et al. 2022; Woolway et al. 2022). As a result of warming air temperatures, altered precipitation, and increased nutrient loads, freshwaters are exhibiting increased variability and degradation, exemplified by the changing occurrence of phytoplankton blooms and their associated negative effects on water quality (Ho et al. 2019; Smucker et al. 2021; Merder et al. 2023). Here, we define a bloom as an aggregation of phytoplankton biomass that can pose water quality risks (adapted from Smayda 1997; Isles and Pomati 2021). Phytoplankton blooms are ubiquitous in many regions (e.g., Coffer et al. 2021; Handler et al. 2023), and it is expected that their occurrence and duration may increase in response to global change (Paerl and Huisman 2009; Paerl and Paul 2012; Chapra et al. 2017; Burford et al. 2020). While some lakes have not exhibited major changes in blooms to date (Kraemer et al. 2017; Wilkinson et al. 2022), other lakes experiencing warming have demonstrated increases in peak summer bloom intensity (Ho et al. 2019), and both temperate and tropical waterbodies are exhibiting increased dominance of cyanobacteria, a major bloom-forming group of phytoplankton (Kosten et al. 2012; Taranu et al. 2015).

Changes in phytoplankton bloom incidence and magnitude due to climate change pose major challenges for drinking water managers because of the ecosystem and health risks caused by toxic blooms. Phytoplankton blooms can produce harmful toxins, odors, and thick surface scums that alter lake water temperature and light availability (Robarts and Zohary 1984; Watson et al. 2016a; Chorus and Welker 2021; Zhang et al. 2022). As phytoplankton biomass decomposes in the water column and at the sediments, oxygen concentrations can decrease (Watson et al. 2016b; Dugener et al. 2023). Low oxygen, in turn, can lead to fish kills, elevated concentrations of nutrients and contaminants, greenhouse gas concentrations, and other degradation (Mortimer 1941; Rysgaard et al. 1994; Bartosiewicz et al. 2019; Hounshell et al. 2021; Carey et al. 2022b). Importantly, drinking or swimming in bloom-contaminated water can cause gastrointestinal and neurological damage and even death for humans, livestock, and pets (Jochimsen et al. 1998; Carmichael et al. 2001; Chorus and Welker 2021). Consequently, given the severity of these threats, water management and decision-making would greatly benefit from knowing a priori when and where a bloom will occur.

Variability in freshwater ecosystem dynamics due to climate change makes it challenging to use historical conditions as a baseline to predict whether a bloom will occur in future. Fundamentally, phytoplankton blooms are emergent phenomena, governed by the complex interactions between individual phytoplankton cells and the surrounding environment (Reynolds 2001; Reynolds and Elliott 2012; Breier et al. 2018; Isles and Pomati 2021). Phytoplankton growth rates can exponentially increase in response to the onset of warm temperatures and high light conditions (as well as other environmental cues), resulting in rapid accumulation of biomass (Reynolds 2006). Similarly, bloom collapse, which can also be triggered by changing environmental conditions, cell senescence, viruses, and other factors, can result in a crash of the phytoplankton populations comprising a bloom (reviewed by Harris et al. 2024). Together, the complex abiotic and biotic interactions that control the development, maintenance, and collapse of blooms (Litchman 2023) motivate the need for new approaches to phytoplankton management.

Fundamentally, forecasts of phytoplankton blooms can help managers adapt and mitigate their risk (e.g., Wynne et al. 2013; Stumpf et al. 2016; Scavia et al. 2023). Here, we define forecasts as predictions of future conditions with specified uncertainty (Table 1; Lewis et al. 2022). While several real-time freshwater forecasting systems for phytoplankton have been developed to date, none are both automated and quantify uncertainty in their predictions (reviewed by Lofton et al. 2023; Table 1), which is critical for implementation and manager use. If managers have forecasts of phytoplankton blooms in advance, they can implement preemptive interventions to mitigate or prevent the bloom from occurring: e.g., apply algaecides, activate engineered water quality systems, or divert water from other lakes or reservoirs (Carey et al. 2022a). In addition, forecasts of blooms could help managers adapt to blooms: e.g., alter water quality treatment processes, install additional filters, initiate swimming/beach closures, or change staffing schedules. Together, these improvements could build more resilience in drinking water systems and would likely produce substantial economic benefits as blooms cost the U.S. economy > $2 billion per year (Dodds et al. 2009). Consequently, there is a pressing need to develop a scalable phytoplankton bloom forecasting system that successfully predicts future blooms in freshwaters.

**Table 1:** Glossary of terms included in the phytoplankton forecasting framework

Term	Definition	References
Data assimilation	The process of statistically comparing a forecast with observations as they become available to update the forecast model for the next time step in an iterative forecasting workflow	Luo et al. (2011)
Ecological forecast	A prediction of a future ecosystem property (or properties) with quantified uncertainty	Lewis et al. (2022)
Edge computing	A distributed computing architecture in which data processing occurs physically at the site of data collection (vs. in the cloud)	Shi et al. (2016)
Ensemble	A set of forecast model outputs that represent alternative predictions (i.e., ensemble members) with their own likelihoods	Dietze (2017a, b)
Forecast uncertainty	A quantitative estimate of a forecast’s spread contributed by multiple sources (e.g., driver data, initial conditions, parameters, process); often derived from ensemble forecast output	Dietze (2017a, b)
Function-as-a-Service (FaaS)	A serverless cloud computing approach to run applications on-demand	Eyk et al. (2017)
Human-centered design	A user-oriented research workflow that integrates human perspectives throughout product development	Kurosu (2011)
Individual-based model (IBM)	Models that simulate the dynamics of interacting individuals, represented by functional relationships and a set of traits	Bonabeau (2002)
Prediction	A quantitative hypothesis of future conditions based on model output	Luo et al. (2011)
Real-time	Integrating data into the forecast workflow soon after the data are collected (usually enabled by the use of automated sensors, wireless data transfer, and cyberinfrastructure)	Carey et al. (2022a)
Surrogate model	A statistical model that emulates a more complex simulation model and can run more quickly, correct bias, and quantify uncertainty	Gramacy (2020)

**Fig. 1:**
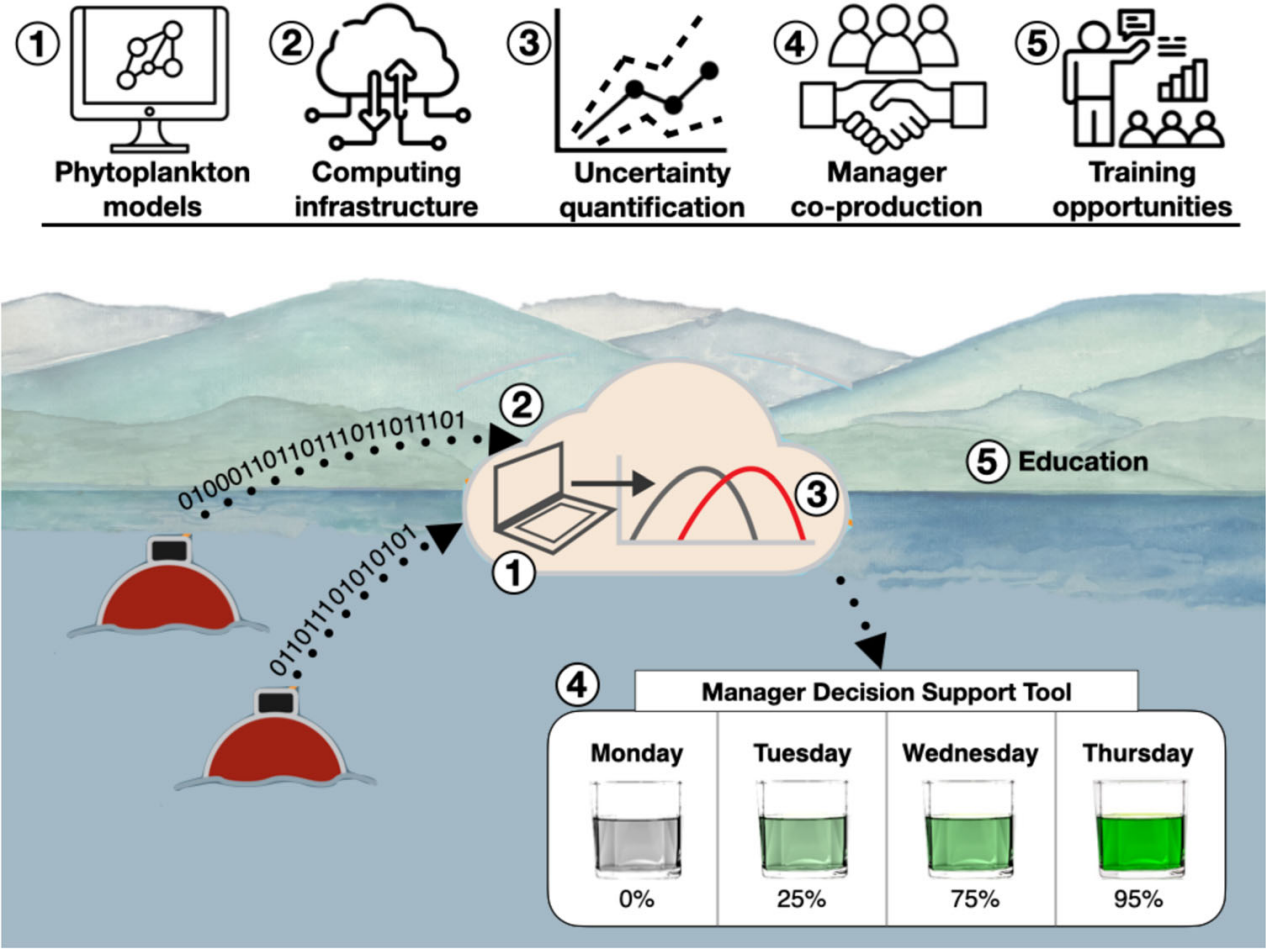
Overview of the five challenges that need to be addressed to build real-time lake phytoplankton forecasting systems: (1) phytoplankton models that are able to predict emergent bloom phenomena; (2) cyberinfrastructure that enables the automated execution of computationally efficient forecasting workflows; (3) robust quantification of forecast uncertainty; (4) co-production of forecast decision support tools with managers; and (5) forecasting training opportunities. The five challenges are superimposed on a conceptual representation of an automated end-to-end forecasting workflow in which lake water quality data are seamlessly transferred via cyberinfrastructure to the cloud for ensemble modeling and uncertainty quantification, and then translated into decision support tools tailored for water managers. Overcoming all of these challenges requires education and training for both forecast users and developers so that forecasts can be optimally created and implemented for decision support. Background reservoir art by K. D. Hamre; Icons were accessed from the Noun Project (Artists: Abd Majd, Evecons Workshop, SeeMoo, cryptocurrency, and Vior)

To be most useful for managers seeking phytoplankton bloom adaptation and mitigation options in the face of climate change, forecasts need to be generated with sufficient advance warning, be updated in real-time as new phytoplankton observations become available, and be translated into useful decision support tools. However, five major challenges remain before such a near-term (e.g., one day to one season-ahead) forecasting system can be realized (Fig. 1). First, most phytoplankton models are currently unable to accurately predict historical or future bloom dynamics (e.g., Flynn 2005; Franks 2009; Fennoochi et al. 2019; Ralston and Moore 2020). Second, setting up and managing the cyberinfrastructure for automated, real-time forecasting requires substantial computing time and resources (Carey et al. 2022a). Third, by definition, forecasts need to have quantified uncertainty (Table 1), which can come from many different sources (e.g., model driver data, parameters), yet is not commonly included in forecasting systems (reviewed by Lewis et al. 2022; Lofton et al. 2023). Fourth, despite their promise, forecasts have yet to be broadly integrated into adaptive management workflows and be used for decision support (Lewis et al. 2022), necessitating new approaches for ensuring that forecasts are useful for managers. Finally, as ecological forecasting is an emerging field (Woelmer et al. 2021), there is a pressing need for forecasting training opportunities, both for researchers developing forecasting systems and for water managers who may be more familiar with reactive (vs. preemptive) decision-making.

Our research team identified these five challenges from our experiences co-developing the FLARE (Forecasting Lake And Reservoir Ecosystems; flare-forecast.org) water quality forecasting system during the past seven years (Thomas et al. 2020; Carey et al. 2022a). While FLARE has primarily been applied to generate lake and reservoir water temperature forecasts to date (e.g., Thomas et al. 2023; Wander et al. 2024), we describe some major hurdles that will need to be overcome to successfully develop phytoplankton forecasting systems that are broadly scalable and useful for water managers.

In this Perspective article, we propose a research agenda for advancing phytoplankton forecasting for freshwater management that addresses each of these five challenges. Our overarching goal is to provide recommendations for how these challenges could be potentially overcome to develop a real-time, automated water quality forecasting system. We note that our proposed approaches may not be appropriate for every forecasting application, and that a diversity of forecasting methods are needed to create an operational lake phytoplankton forecasting system for waterbodies across the globe (akin to the well-established meteorological forecasting systems that generate real-time weather forecasts in many countries). However, while such a global system likely remains several years to decades away, we forecast that the deployment of real-time, automated phytoplankton forecasts with specified uncertainty on select lakes and reservoirs is well within our grasp and possible on much shorter-term horizons.

## Recommendations

### Modeling needs for forecasting phytoplankton

Blooms remain notoriously hard to predict via a range of modeling approaches, despite a century of research by aquatic scientists aiming to improve water quality management (Fig. 1, Challenge 1; Flynn 2005, Ralston and Moore 2020, Rousso et al. 2020, Litchman 2023). Both simple *and* complex models struggle to accurately predict blooms (reviewed by Rousso et al. 2020). For example, many current trait-based approaches to phytoplankton modeling apply a small, static set of known functional relationships (e.g., nutrient uptake rates and temperature-dependent growth rates) to aggregated groups of phytoplankton in deterministic process-based models that range in complexity from simple nutrient uptake equations to coupled hydrodynamic-water quality models (Franks 2009; Page et al. 2018; Litchman 2023). While these models can reproduce median phytoplankton levels (e.g., Elliott et al. 2006; Page et al. 2018; Soares and Calijuri 2021), they often fall short of predicting emergent bloom events because aggregating heterogeneous species into one group neglects the inherent variability of individual cell responses to the functional relationships (Bruggeman and Kooijman 2007; Hellweger et al. 2008; Cropp and Norbury 2009; Cottingham et al. 2021). This shortcoming of modeling phytoplankton as aggregated functional groups motivates the need to model these complex dynamics at the individual cell level to predict blooms (Ranjbar et al. 2021). Similarly, while machine learning models are showing increasing promise at predicting phytoplankton blooms (Gupta et al. 2023; Lin et al. 2023; Silva et al. 2023), more work is needed to robustly quantify uncertainty and incorporate scientific knowledge into these models to maximize their utility for water management decision-making (Abdar et al. 2021; Varadharajan et al. 2022; Lapeyrolerie and Boettiger 2023). We refer readers to reviews by Rousso et al. (2020) and Lofton et al. (2023) on the diversity of models currently being used to predict and forecast phytoplankton blooms in lakes, as well as their relative predictive skill, advantages, and shortcomings.

In response to this challenge, we propose three key characteristics of potential forecast modeling approaches that show great promise for improving bloom prediction. First, individual-based models (IBMs) are a powerful simulation approach specifically designed to study collective phenomena (e.g., phytoplankton blooms) that emerge from functional relationships implemented to govern the behavior of individuals (Table 1; Bonabeau 2002). Each individual in an IBM is assigned a set of traits (e.g., age, size) by drawing from a distribution of possible trait values (DeAngelis and Grimm 2014), thereby explicitly accounting for heterogeneity among individuals in how they respond to the functional relationships (e.g., Engel et al. 2017). In addition, the behavior of individuals and the relative importance of different processes represented in the model can vary based on environmental conditions (Simon and Fortin 2020) or the number of individuals present at a site (Ellis and Petrovskaya 2020), permitting both abiotic variables and population/community interactions to drive individual dynamics over time and space. Finally, individuals can have a memory of past conditions, which can dynamically alter individual behavior. For example, alteration of the photosynthesis rate of a phytoplankton cell due to changes in light availability will alter the cell’s carbon content and, in turn, change the cell’s sinking rate in the lake (Feng et al. 2018). Together, these IBM attributes may help improve our ability to forecast emergent bloom phenomena (Ranjbar et al. 2021), though we note that IBMs have yet to be applied for real-time forecasting applications and may not be appropriate for all modeling contexts. To best evaluate IBM performance for bloom forecasting, a suite of evaluation metrics should be used that include estimates of forecast accuracy (e.g., bias, root mean square error; Joliffe and Stephenson 2012), forecast precision (e.g., reliability of forecast confidence intervals, Bröcker and Smith 2007), combined accuracy and precision (ignorance score, continuous ranked probability score; Gneiting et al. 2005; Smith et al. 2015), and forecast skill (e.g., skill scores calculated by comparing the forecast vs. null models used for forecast benchmarking; Pappenberger et al. 2015).

Second, it is likely that we need to use an ensemble modeling approach to improve the performance of phytoplankton forecasts. Ensemble modeling (see Table 1) involves generating a set of model simulations (ensemble members) that can differ in their starting conditions, model parameter values, process uncertainty (represented by adding random noise), model inputs (e.g., inputs from an ensemble weather model or hydrological model), and/or model structures (e.g., different types of models; Dietze 2017a). Ensemble modeling is particularly powerful for simulating the dynamics of non-linear systems (Smith 2001), such as phytoplankton blooms, where ensemble members can diverge widely in response to small differences in model initial conditions, driver data, parameters, and process/stochastic noise. When applied to IBMs, members of an ensemble can also represent different distributions of parameters describing traits (e.g., phytoplankton cell size, sinking rates; Litchman et al. 2010; Cocucci et al. 2022; Laraib et al. 2023). Thus, because IBMs inherently have multiple ensemble members with associated relative likelihoods (i.e., individuals), IBMs may be able to better quantify both the probabilities of a large range of phytoplankton bloom outcomes and their uncertainty (sensu Ralston and Moore 2020), relative to other model types.

Third, data assimilation, i.e., the updating of models with observations when they become available (Table 1), will also likely improve model performance (reviewed by Luo et al. 2011; Niu et al. 2014). Data assimilation has been found to greatly improve water quality model performance in a suite of other lake studies, including for methane emissions (McClure et al. 2021), organic carbon (Zwart et al. 2019), and water temperature (Thomas et al. 2020). Data assimilation is important for bloom forecasting because the uncertainty generated by the ensemble will likely be large and data assimilation can help filter out the ensemble members (and their associated parameters, states, and traits) that are inconsistent with the most recent observations (e.g., Woelmer et al. 2022). This filtering reduces the ensemble spread when forecasts are initiated, thus reducing forecasting uncertainty (Wander et al. 2024). As phytoplankton conditions can change rapidly in response to environmental variability, the integration of new data in near-real time (e.g., over the past day) to update model states, parameters, and initial conditions can provide an important ‘check’ on forecast model trajectories and allow the model to evolve in response to fluctuations in phytoplankton levels. While the application of data assimilation in freshwater modeling studies is increasing, its use for water quality forecasting is still limited (reviewed by Lofton et al. 2023), and we encourage interested readers to see reviews on data assimilation by Cho et al. (2020) and Lahoz and Schneider (2014).

### Building the computational workflows for real-time forecasting

A second major challenge for phytoplankton forecasters is cyberinfrastructure and computational efficiency (Fig. 1, Challenge 2). Real-time daily forecasting entails downloading observations from phytoplankton sensors in the lake, using those data to update models (i.e., data assimilation) to run the many different simulations comprising an ensemble forecast into the future, and then disseminating the forecast to managers, all within 24 h before the next day’s forecast needs to be generated (Carey et al. 2022a). For forecasts to run automatically, data need to be transferred securely and wirelessly from sensors deployed in the field to models that are running in near-real time (Daneshmand et al. 2021). This automation requires the coordinated execution of a distributed workflow encompassing various hardware and software modules that are responsible for sensor data collection and staging, reliable data transfers, data quality assurance/quality control (QA/QC), ensemble model execution, and data summarization and visualization for use by managers (Thomas et al. 2020). In general, forecasting workflows require computation taking place at both well-resourced cloud computing data centers (which have massive servers and storage), as well as at the network’s edge, i.e., sensors deployed in lake sites that have limited network connectivity and power (Shi et al. 2016), which can be challenging to orchestrate. Moreover, the computational demands of the phytoplankton forecasting models need to be manageable within the overall end-to-end workflow: e.g., a reason why IBMs are not currently used for real-time, daily water quality forecasting is because of their high computational cost (Ranjbar et al. 2021), resulting in IBMs with computational times exceeding simulation durations.

A combination of edge and cloud computing using the Function-as-a-Service (FaaS; Eyk et al. 2017) paradigm may provide a new approach for facilitating data transfer and automation of end-to-end phytoplankton forecasting workflows (see Table 1). Edge computing is a distributed computing architecture in which data processing occurs physically at the site of data collection, thereby decreasing computing, storage, and connectivity needs (Shi et al. 2016), and FaaS is a serverless computing approach to run event-driven applications on-demand (Table 1; Eyk et al. 2017; Schleier-Smith et al. 2021). Two key benefits of FaaS are its scalability and its capacity to abstract server deployment and management without needing constant user input (hence the term “serverless”; Eismann et al. 2021; Schleier-Smith et al. 2021). FaaS is typically supported on cloud resources for scalable execution, but the platform is also extendable to edge resources for event-driven computation (Mampage et al. 2022).

To apply an event-driven and scalable FaaS platform to lake phytoplankton forecasting, an end-to-end ensemble forecasting workflow would first be divided into functions. At the waterbody being forecasted, FaaS functions could include accessing data from environmental sensors deployed at a lake and its inflowing streams; processing data for QA/QC; transferring data to cloud storage via edge gateways, or mini-computers deployed in the field (Daneshmand et al. 2021); and initiating IBM simulations. Each function would be invoked by an event (e.g., a daily timer, or another trigger, such as the completion of the preceding function in the workflow). To forecast rapidly changing phytoplankton conditions, a FaaS platform could be deployed at the edge gateway to both detect when phytoplankton are increasing in the lake and react by triggering functions that implement an increased frequency of sensor data collection and forecast updating. Importantly, these actions could be automatically triggered where the data are collected (i.e., by the sensors at the edge), without any human intervention. While FaaS platforms have primarily been tested in commercial cloud environments (Lynn et al. 2017) and scientific computing applications (Li et al. 2022), applying a FaaS system to adaptively run phytoplankton forecasting workflows spanning both edge and cloud computing could set the foundation for its deployment in other environments. Helpful resources for learning more about FaaS and its many potential applications include Schleier-Smith et al. (2021) and Shafiei et al. (2022).

To improve the computational efficiency of the end-to-end forecasting workflow, surrogate modeling, a statistical computing technique, could enable researchers to harness the power of IBMs and other highly parametrized models for real-time, daily phytoplankton forecasts (Tsattalios et al. 2023). Surrogate modeling, or emulation, is the enterprise of meta-modeling a simulation model, which has many advantages (Table 1; Gramacy 2020). The most well-known benefit of a surrogate model is computational speed (Sacks et al. 1989). By abstracting a simulation with statistical distributions, thereby “memorizing” the input–output relationship exhibited by the computationally intensive model, the surrogate can quickly mimic the essence of the simulation model without having to run the model. While surrogates have yet to be applied to emulate phytoplankton IBMs, they have been successfully used to emulate IBMs of wastewater microbial communities (Oyebamiji et al. 2017). For this application, the surrogate increased computational efficiency by 220-fold, decreasing simulation model run times from 6 h to < 2 min (Oyebamiji et al. 2017), suggesting that surrogates could enable phytoplankton IBMs to be integrated into real-time, daily forecasting workflows in the future.

### Forecasts are inherently uncertain

Third, to the best of our knowledge, we are unaware of any automated, near-term freshwater phytoplankton forecasting systems that quantify uncertainty in predictions (Lofton et al. 2023), which may be because robust forecast uncertainty quantification is challenging (Fig. 1, Challenge 3; Dietze et al. 2018). Following our definition of a forecast (Lewis et al. 2022), “forecasts” without specified uncertainty are simply predictions (Lofton et al. 2023). Out of 83 papers published between 2017 and 2022 that presented future water quality predictions, the majority (64%) did not incorporate uncertainty (reviewed by Lofton et al. 2023). If uncertainty is not included in a “forecast,” managers will be unable to interpret the risk associated with a prediction of a phytoplankton bloom, potentially altering subsequent decisions (Berthet et al. 2016). For example, a forecast with a 95% probability that phytoplankton levels will exceed a drinking water threshold next week will likely trigger different management decisions than a forecast with a 5% probability of exceeding the threshold. Moreover, if uncertainty is not specified in a “forecast,” managers may assign their own uncertainty estimates to predictions, which could be incorrect or distorted by internal biases (Berthet et al. 2016). Users may also underestimate probabilities of adverse events, thereby increasing risks of human health or recreational impacts, or overestimate probabilities, leading to increased costs (Roberts et al. 2008).

There are many different sources of uncertainty in forecasts, including uncertainty in model parameters, initial conditions, processes, and driver data, among others (Dietze 2017a; Jakeman et al. 2019; Geary et al. 2020). Of “true” freshwater forecasts that incorporate uncertainty, most include only one or two sources of uncertainty (e.g., Olsson et al. 2024), and those forecasts that do include more than one source rarely partition the individual contributions of their uncertainty sources to total forecast uncertainty (Lofton et al. 2023). As a result, the relative importance of model parameters, initial conditions, processes, and driver data to total uncertainty in water quality forecasts is not well known (Lofton et al. 2023). Quantifying the relative contribution of individual uncertainty sources ensures that the most important sources of total uncertainty are accounted for in a forecast, resulting in well-calibrated uncertainty estimates (Olsson et al. 2024), and identifies how best to reduce total forecast uncertainty (Dietze 2017b; Lofton et al. 2022).

Consequently, researchers need to carefully consider how best to quantify uncertainty as they develop phytoplankton forecasts. Ensemble modeling is a common approach for quantifying forecast uncertainty, whereby multiple alternate simulations of the forecast model are run with different model driver datasets, parameter values, initial conditions, and/or process/stochastic noise (Table 1; Dietze 2017a). The total uncertainty in a forecast can then be determined by simultaneously representing each of these sources in the ensemble, with the individual uncertainty sources and their interactions summing to total forecast uncertainty (Dietze 2017a). When using this ensemble modeling approach, driver data uncertainty may be the most straightforward uncertainty source to incorporate because ensemble members from e.g., weather forecast or hydrological models can be directly used as inputs to different ensemble members of a forecasting model (e.g., Dietze 2017a; Thomas et al. 2020; Zwart et al. 2023; Olsson et al. 2024). Parameter and process uncertainty can also be straightforward to estimate when using simple statistical models (e.g., regression time series models; McClure et al. 2021; Lofton et al. 2022; Woelmer et al. 2022) because the probability distributions of the parameters and residual error (which represents process uncertainty) in these simple models can be easily sampled from and applied to forecast ensemble members (Dietze 2017a). Parameter and process uncertainty may be more challenging to quantify when using complex process-based models that include many equations, model states, and parameters (e.g., hydrodynamic-water quality models and IBMs), highlighting the value of Bayesian approaches for estimating probability distributions (Hobbs and Hooten 2015). While Bayesian approaches can be used to quantify parameter and process uncertainty, they are computationally intensive, requiring simplifications to generate robust estimates of uncertainty for real-time, daily forecasting. Thus, surrogate models (see above) make it more computationally efficient and feasible to quantify the dominant sources of forecast uncertainty in IBMs and other similar types of models (Gramacy 2020), thereby helping to both prioritize improvements to phytoplankton forecasting systems (Lofton et al. 2022) and help managers better interpret the risk of potential future blooms (Woelmer et al. 2023).

### Co-producing forecasts with managers for decision support

Fourth, forecasts are extraordinarily challenging to communicate because there are many different ways to report forecast uncertainty, resulting in contradictory and frequently incorrect interpretations by users (Fig. 1, Challenge 4; Broad et al. 2007, Ramos et al. 2013, Cheong et al. 2016, Kinkeldey et al. 2017, Mulder et al. 2023). For example, multiple depictions of uncertainty in phytoplankton bloom forecasts are possible (e.g., time series of phytoplankton biomass with widening predictive intervals into the future, percent likelihood of future exceedance of a certain bloom threshold) and each visualization has different possible (but unknown) interpretations by water managers (Woelmer et al. 2023). While many studies have explored different approaches for communicating uncertainty in scientific data (e.g., Olston and Mackinlay 2002; Smith Mason et al. 2017; Wiggins et al. 2018), determining how best to translate uncertainty in forecasts into visualizations for decision-makers is unresolved, even for well-studied weather forecasts (Gerst et al. 2020; Kamal et al. 2021). Managers likely need a suite of different visualizations to guide different decisions, but the best practices for developing useful forecast output are unknown (Bodner et al. 2021). Moreover, it remains unclear how water managers interpret model outputs or operationalize data into management decisions, creating challenges for researchers trying to identify and develop the most useful forecasting models and visualizations.

Consequently, forecasters must work closely with managers when developing phytoplankton forecasting systems to ensure that forecast output is usable by decision-makers (Bodner et al. 2021; Carey et al. 2022a; Lofton et al. 2023). Many environmental models and visualizations have failed to be adopted by end users because they have not captured relevant trade-offs and have been perceived by practitioners or managers as inconsistent with their experience and expertise (Jakeman et al. 2006; McIntosh et al. 2008; Dubois et al. 2020). Ultimately, the co-production of forecasts using human-centered design (Table 1) takes time and requires iterative interaction of researchers and managers to evaluate the forecasting system, learn from its successes and failures, and update the system to improve decision-making based on forecasts (Chun and Conley 2011; Clark 2011; Boy 2012).

As part of forecasting system co-production, it would be useful for researchers to elicit managers’ (1) operational metrics of phytoplankton blooms; (2) learn managers’ perceptions of the ecological, human health, and technical hazards linked to phytoplankton blooms that drive decision-making in their water management; and (3) assess managers’ attitudes toward uncertainty in forecasts of phytoplankton blooms. For example, if a focal group of water managers make treatment decisions based on algal cell count data, then a forecast of chlorophyll-a may not be easily interpretable or useful. Likewise, managers of a lake with swimmers may quantify a bloom based on the areal extent of a surface scum, whereas managers of a lake where drinking water is extracted from deep intakes may quantify a bloom based on biomass concentrations throughout the water column. It is likely that managers’ perceptions of the hazards associated with phytoplankton blooms also influence how they respond to different representations of uncertainty in bloom forecasts, so knowing this information early in the design process can help researchers present uncertainty in forecast visualizations more effectively (e.g., via predictive intervals on a time series graph, a risk index, etc.). Overall, manager engagement throughout forecasting system development is essential for creating phytoplankton forecast models and decision support tools that are useful.

### Forecasting training for both researchers and managers

Fifth, there is a pressing need for forecasting training opportunities, both for researchers developing forecasting systems and for water managers who may be more familiar with reactive (vs. preemptive) decision-making (Fig. 1, Challenge 5). For the researchers, developing phytoplankton forecasts requires skills in programming, statistics, ecosystem modeling, uncertainty analysis, data visualization, cyberinfrastructure, and decision science, which are rarely taught simultaneously in many undergraduate and graduate training programs (Woelmer et al. 2021; Willson et al. 2023). For the managers, water treatment curricula generally lack training on how to interpret high-frequency monitoring data and forecasts to guide predictive management interventions (e.g., VAC 2017). The lack of training in these key forecasting skills hinders the development of phytoplankton forecasting systems and their use for decision support.

Environmental models emerge from the natural sciences as tools to evaluate the possible role of alternative mechanisms and modes of action in environmental phenomena of interest. When adapted to a decision context, they frequently fail to capture the full range of physical, engineered, and social trade-offs and feedbacks that constrain decision-makers (Calder and Schartup 2023). Meanwhile, managers may seek high-level information about operational strategies to achieve environmental objectives but may have less knowledge about the network of phenomena that govern outcomes of interest (Calder et al. 2020). Therefore, iterative model development with both researchers and practitioners is crucial for creating models that increase the scientific basis of decision-making while addressing management realities and constraints.

Together, we need to create training opportunities for both researchers *and* managers. This training could take many forms, tailored for each community but developed with complementary learning objectives to facilitate collaboration between the two groups. For example, training for the managers could include short educational modules focused on key forecasting concepts embedded within manager certification programs, as well as workshops at management-focused conferences. These modules could then be adapted for undergraduate and graduate student courses and programs and include an additional focus on the data science skills underlying forecast development. Ultimately, managers need to have a high-level understanding of how forecasts are developed and their key assumptions (e.g., how forecast uncertainty is being quantified) and researchers need to understand managers’ needs and how decision-making occurs to ensure that forecasts are useful (e.g., which bloom adaptation and mitigation opportunities are available). Moreover, both groups need to be able to interpret probabilistic forecast visualizations and evaluate different representations of uncertainty. Overall, while manager training has not yet been implemented into ecological forecasting training to date (e.g., Moore et al. 2022; Willson et al. 2023; Woelmer et al. 2023), we encourage researchers to think carefully about how best to craft educational materials that can be used to train the next generation of both forecast developers and users together.

## Conclusions

Here, we outline five major challenges that currently serve as bottlenecks for developing real-time phytoplankton bloom forecasting systems. We focused specifically on these five challenges because they inherently require interdisciplinary expertise to be overcome, and may be overlooked by researchers as they embark on forecasting system development. We note that there are many other thorny (i.e., exciting!) challenges that also need to be addressed: e.g., accessing lake data for assimilation from sites without in situ observations, potentially via satellite imagery (Schaeffer et al. 2024); developing accurate forecasted driver data needed to run phytoplankton models (e.g., hydrology, water chemistry); developing the cyberinfrastructure to support real-time ensemble forecasting workflows for many water quality variables simultaneously; extending phytoplankton forecast horizons to improve the usability of forecasts for decision-making (e.g., Jackson-Blake et al. 2022); and maintaining forecasting systems sustainably over the long-term (Carey et al. 2022a). We also note that our proposed solutions to the five challenges may not be applicable for all forecasting contexts, and that forecasting may not be appropriate for all management needs (e.g., Hobday et al. 2019). Rather, given the enormity of the task of setting up a real-time automated forecasting system, we hope that our recommendations serve as a catalyst for helping forecasters examine which of these approaches might be the right fit (or not) for their needs, and aim to lower the barrier to their implementation. Ultimately, phytoplankton bloom forecasts hold much promise for expanding the toolbox of options for water managers. Given the urgent need for new approaches to phytoplankton bloom management in the face of climate change, we encourage researchers and managers alike to consider forecasting as a solutions-focused approach for climate change adaptation and mitigation.
